# Comparative genomic analysis of plasmids harboring *bla*_OXA-48_-like genes in *Klebsiella pneumoniae*

**DOI:** 10.3389/fcimb.2022.1082813

**Published:** 2022-12-20

**Authors:** Wang Li, Hengzhao Guo, Yi Gao, Xiaofan Yang, Ruirui Li, Shuangyu Li, Chunlong Sun, Wen Du, Shaopeng Chen, Pengpeng Xu, Wenwen Huang, Jia Shi, Xinfeng Yi, Xiaobin Li

**Affiliations:** ^1^ Shandong Provincial Engineering and Technology Research Center for Wild Plant Resources Development and Application of Yellow River Delta, College of Biological and Environmental Engineering, Binzhou University, Binzhou, China; ^2^ Binzhou Key Laboratory of Chemical Drug R&D and Quality Control (preparation), Binzhou, China; ^3^ Department of Radiation Oncology, Zhuhai People’s Hospital (Zhuhai hospital affiliated with Jinan University), Zhuhai, China; ^4^ Department of Stomatology, Zhuhai People’s Hospital (Zhuhai hospital affiliated with Jinan University), Zhuhai, China; ^5^ Department of Neurosurgery, Zhuhai People’s Hospital (Zhuhai hospital affiliated with Jinan University), Zhuhai, China; ^6^ Zhuhai Precision Medical Center, Zhuhai People’s Hospital (Zhuhai hospital affiliated with Jinan University), Zhuhai, China

**Keywords:** *Klebsiella pneumoniae*, plasmid, *bla*_OXA-48_-like, conjugative transfer region, genetic context

## Abstract

The emergence and spread of carbapenem-resistant *Klebsiella pneumoniae* (CRKP) is a serious medical problem worldwide. Acquired OXA-48-like carbapenemases encoded by plasmids are important causes of carbapenem resistance in *K. pneumoniae*. To explore the links between plasmids and *bla*_OXA-48_-like genes in *K. pneumoniae*, we systematically analyzed the variants of *bla*_OXA-48_-like plasmid replicon types, phylogenetic patterns, geographic distribution, conjugative transfer regions, and the genetic environments surrounding *bla*_OXA-48_-like of 191 *bla*_OXA-48_-like-harboring plasmids, which were identified from 4451 plasmids of *K. pneumoniae* downloaded from GenBank. Our results showed that seven different variants of *bla*_OXA-48_-like genes were identified from the 191 *bla*_OXA-48_-like-harboring plasmids in *K. pneumoniae*, with *bla*_OXA-48_, *bla*_OXA-232_, and *bla*_OXA-181_ being highly prevalent. In *K. pneumoniae*, *bla*_OXA-48_ was mainly carried by the composite transposon Tn*1999.2* located on IncL/M-type conjugative plasmids, which were mainly geographically distributed in Switzerland, Germany, and China. In *K. pneumoniae*, the *bl*a_OXA-232_ gene was mainly carried by 6.1-kb ColKP3-type mobilizable plasmids, which were mainly isolated in India. In *K. pneumoniae*, *bla*_OXA-181_ was mainly carried by a group of 50-kb ColKP3-IncX3 hybrid conjugative plasmids and a group of small ColKP3-type mobilizable plasmids with lengths of 5.9–9.3 kb, the former was sporadically discovered in China, South Korea, India, and Czech Republic, while the latter was almost all isolated in India. In addition, five *bla*_OXA-245_-harboring 65.9-kb IncL plasmids of *K. pneumoniae* isolated in Spain were found to have the genetic context of *bla*_OXA-245_ more complicated than that of *bla*_OXA-48_-harboring IncL/M-type plasmids, with two copies of IS*1R* inserted both upstream and downstream of *bla*_OXA-245_-*lysR*. These findings enhance our understanding of the genetic diversity of *bla*_OXA-48_-like-harboring plasmids in *K. pneumoniae*.

## 1 Introduction

The rapid increase in carbapenemase-producing *Enterobacterales* has become a threat to public health (Kim et al., 2021). OXA-48-like carbapenemases are important causes of carbapenem resistance in *Enterobacterales* (Pitout et al., 2019) and consist of 261–265 amino acids (Poirel et al., 2004). Among the various OXA-48-like carbapenemases, OXA-48, OXA-181, OXA-232, OXA-204, OXA-162, and OXA-244, in that order, are the most common. Surveillance studies based on molecular methodologies have indicated that OXA-48-like carbapenemases remain the second or third most common carbapenemases in *Enterobacterales* globally (de Jonge et al., 2016; Karlowsky et al., 2017; Han et al., 2020). Notably, *Enterobacterales* producing OXA-48-like carbapenemases are endemic in certain parts of the world (Pitout et al., 2019). OXA-48-like carbapenemases are widely distributed among members of *Enterobacterales*, mainly *Klebsiella pneumoniae* isolated from hospital sites (de Jonge et al., 2016; Karlowsky et al., 2017; Han et al., 2020).

*K. pneumoniae* is a significant cause of both community- and hospital-acquired infections such as pneumonia, urinary tract infections, bloodstream infections, and septicemia (Pitout et al., 2015; Bengoechea and Sa Pessoa, 2019) and is known for its high frequency of antimicrobial resistance (AMR) genes (Navon-Venezia et al., 2017; Wyres and Holt, 2018). *K. pneumoniae* has been classified into one of the ESKAPE pathogens, which are the leading cause of nosocomial infections (Pandey et al., 2021). The emergence and spread of carbapenem-resistant *K. pneumoniae* (CRKP) have become a severe medical problem worldwide (Navon-Venezia et al., 2017). AMR in CRKP isolates is frequently encoded by plasmid-borne genes that can disseminate horizontally (Rozwandowicz et al., 2018).

Plasmids are autonomously replicating extrachromosomal DNA molecules in many bacterial strains (Le Roux et al., 2011), which are the key element in horizontal gene transfer in the microbial community (Fang and Zhou, 2020). According to the transferability of plasmids, they can be classified into the non-transferable plasmids, conjugative (self-transferable) plasmids and mobilizable plasmids (Branger et al., 2018). For CRKP isolates, conjugative plasmids are important vehicles for the spread of AMR genes (Smillie et al., 2010; Ravi et al., 2018). Such plasmids typically have a conserved backbone and variable accessory regions (Brown et al., 2013; Sitter et al., 2021). The conserved backbone region includes genes responsible for plasmid-related traits, *e.g.*, replication regulation and conjugation functions. The variable accessory region is loaded with accessory genes, such as AMR genes, usually located on transposons or integrons (Norman et al., 2009; Norberg et al., 2011). For the conjugative plasmids, the transfer regions responsible for conjugation typically comprise four key modules: origin of transfer (*oriT*) site, relaxase gene, gene encoding type IV coupling protein (T4CP), and gene cluster for type IV secretion system (T4SS) (de la Cruz et al., 2010). Notably, mobilizable plasmids are also important contributors to the dissemination of AMR genes, usually carrying indispensable *oriT* sites and a limited amount of *mob* genes, which cannot transfer independently but can be mobilized by conjugative elements (Ramsay and Firth, 2017).

Currently, studies on the comprehensive analysis of *bla*_OXA-48_-like-harboring plasmids and their conjugative transfer regions in *K. pneumoniae* are limited. In this work, we performed *in silico* characterization and comparative analysis of *bla*_OXA-48_-like-harboring plasmids of *K. pneumoniae* using bacterial plasmids available in the NCBI GenBank database. We systematically analyzed the variants of *bla*_OXA-48_-like, replicon type, phylogenetic pattern, conjugative transfer region, and genetic context of *bla*_OXA-48_-like-harboring plasmids of *K. pneumoniae*.

## 2 Materials and methods

### 2.1 Plasmid sequences from the NCBI database

A total of 4451 plasmids of *K. pneumoniae* (**Table S1**) were downloaded from the GenBank (Benson et al., 2018) genome database (https://www.ncbi.nlm.nih.gov/genome/browse/#!/plasmids/815/) on April 26, 2022. The DNA files in FASTA format were downloaded in batches into our server built on the Linux-CentOS7 operating system using two Bioperl modules: Bio::DB::GenBank and Bio::SeqIO.

### 2.2 Determination of *bla*_OXA-48_-like-harboring plasmids of *K. pneumoniae*

The acquire antimicrobial resistance genes (ARGs) of plasmids were determined using the software ResFinder standalone version 4.1 (Bortolaia et al., 2020), with a minimum identity of 90%, a minimum coverage of 60%. The term “*bla*_OXA_” was used to search in the “Resistance gene” list within the ResFinder results to determine the *bla*_OXA_-positive plasmids of *K. pneumoniae*. The plasmids harboring *bla*_OXA-48_-like genes were further determined by mapping the “Resistance gene” to file phenotypes.txt of the ResFinder database (Bortolaia et al., 2020) to extract the information column titles termed “Notes” of the file phenotypes.txt. The variants of *bla*_OXA-48_-like genes in some plasmids were not determined using the ResFinder software. They were further analyzed using the CARD database (Alcock et al., 2020) and the beta-lactamase database (Naas et al., 2017).

### 2.3 Geographic location and origin of the *bla*_OXA-48_-like-harboring plasmids of *K. pneumoniae*

Information about geographic location and origin of the *bla*_OXA-48_-like-harboring plasmids in *K. pneumoniae* were extracted in batches from the section “source” of corresponding genomic files in GenBank format, using the key words “country”, “isolation_source” and “host”. For some plasmids with no relevant records on the geographic location and origin, we tried to search the corresponding information from their published papers according to the PubMed ID (PMID) numbers included in the GenBank files. To ensure accuracy of data, only data collected in strict accordance with the above two standards could be included in our analysis.

### 2.4 Replicon sequence analysis of the *bla*_OXA-48_-like-harboring plasmids of *K. pneumoniae*

Plasmid replicon typing of *bla*_OXA-48_-like-carrying plasmids was executed in the PlasmidFinder software (Carattoli and Hasman, 2020). Based on the database “Enterobacteriales,” the genomic files (FASTA format) of *bla*_OXA-48_-like-carrying plasmids were analyzed in batches using the PlasmidFinder software version 2.0.1, with a minimum identity of 95% and a minimum coverage of 60%. The database was updated on November 29, 2021.

### 2.5 Phylogenetic cladogram of the *bla*_OXA-48_-like-harboring plasmids of *K. pneumoniae*

The files of the *bla*_OXA-48_-like-carrying plasmids of *K. pneumoniae* in GenBank format were downloaded in batches using Bioperl (Bio::DB::GenBank and Bio::SeqIO). Using Bioperl/Bio::SeqIO, genomic files containing protein sequences were extracted from the files in GenBank format. Phylogenetic cladogram according to the presence/absence of orthologous gene families of all *bla*_OXA-48_-like-harboring plasmids in *K. pneumoniae* were analyzed. A binary gene presence/absence matrix was built using OrthoFinder (Emms and Kelly, 2019), and then hierarchical clustering was conducted using PAST3 (Hammer et al., 2001) and eventually displayed using iTOL (Letunic and Bork, 2016).

### 2.6 *In silico* characterization of the conjugative transfer regions of *bla*_OXA-48_-like-harboring plasmids

The files (GenBank format) of the *bla*_OXA-48_-like-harboring plasmids in *K. pneumoniae* were analyzed in batches using oriTfinder (Li et al., 2018) (standalone version) to identify the presence/absence of *oriT*s, relaxase genes, T4CP genes, and gene clusters for T4SS. Moreover, the types of *oriT*s, relaxase genes, T4CP genes, and gene clusters for T4SS of the plasmids were determined based on the oriTDB database (Li et al., 2018). In addition, the types of gene clusters for T4SS were classified based on the SecReT4 database (Bi et al., 2013).

### 2.7 Analysis of genetic context of the *bla*_OXA-48_-like genes

The bacterial insertion sequences of the *bla*_OXA-48_-like-harboring plasmids in *K. pneumoniae* were explored using ISfinder (Siguier et al., 2006). Comparisons among the genetic contexts of *bla*_OXA-48_-like genes of the plasmids were carried out using Easyfig (Sullivan et al., 2011) or BLAST Ring Image Generator (Alikhan et al., 2011).

## 3 Results

### 3.1 Variants of *bla*_OXA_ genes in the *bla*_OXA-48_-like-harboring plasmids of *K. pneumoniae*

**Figure 1:**
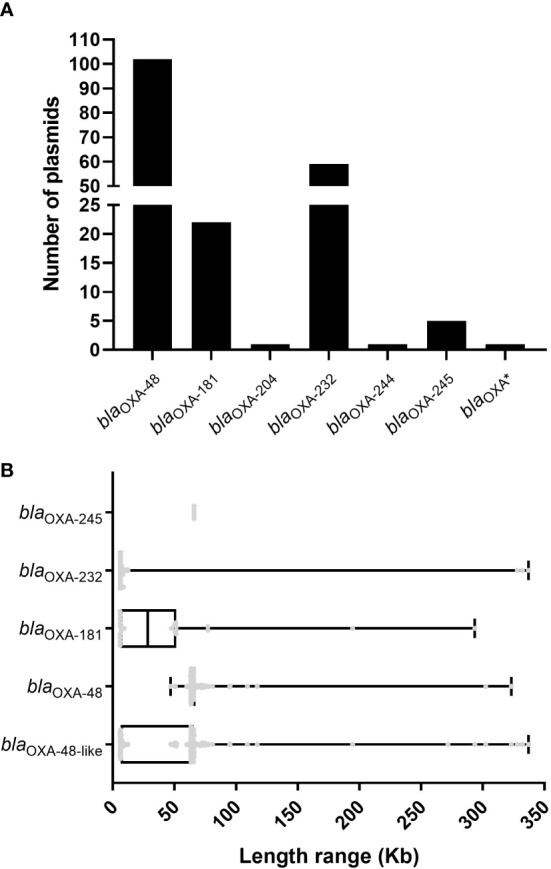
Characteristics of the 191 *bla*_OXA-48_-like-harboring plasmids in *K. pneumoniae*. **(A)** Histogram of number of variants of *bla*_OXA-48_-like genes among the 191 *bla*_OXA-48_-like-harboring plasmids of *K. pneumoniae*. **(B)** Box plot of the length distribution of the five *bla*_OXA-245_-harboring plasmids, 59 *bla*_OXA-232_-harboring plasmids, 22 *bla*_OXA-181_-harboring plasmids, 102 *bla*_OXA-48_-harboring plasmids, and all the 191 *bla*_OXA-48_-like-harboring plasmids of *K. pneumoniae*.

Using ResFinder, 191 (4.29%) *bla*_OXA-48_-like-harboring plasmids (**Table S2**) were identified from 4451 plasmids of *K. pneumoniae* downloaded from the GenBank genome database. Among the 191 *bla*_OXA-48_-like-harboring plasmids of *K. pneumoniae*, 197 *bla*_OXA-48_-like genes belonging to seven *bla*_OXA-48_-like variants were identified. Of these seven variants, *bla*_OXA-48_ was the most dominant, followed by *bla*_OXA-232_ and *bla*_OXA-181_ (**Figure 1A**). A total of 102 plasmids harboring *bla*_OXA-48_ were screened from the 191 *bla*_OXA-48_-like-harboring plasmids, including 100 plasmids with only one copy of *the bla*_OXA-48_ gene in their genomes and two plasmids containing two copies of *bla*_OXA-48_ genes in their genomes (**Table S2**). Of the 59 *bla*_OXA-232_-harboring plasmids, 55 plasmids were found to harbor only one copy of *bla*_OXA-232_ in their genomes, and four plasmids were found to carry two copies of *bla*_OXA-232_ in their genomes) (**Table S2**). All 22 *bla*_OXA-181_-harboring plasmids harbored one copy of the *bla*_OXA-181_ gene in their genomes. In addition, one plasmid harboring *bla*_OXA-204_, one carrying *bla*_OXA-244_, and five *bla*_OXA-245_-harboring plasmids were identified in this study. Meanwhile, one plasmid from *K. pneumoniae* strain LZK001 harbored one *bla*_OXA_ gene, similar to *bla*_OXA-48_ and *bla*_OXA-244_, according to the results of ResFinder.

### 3.2 Lengths of *bla*_OXA-48_-like-harboring plasmids of *K. pneumoniae*

We analyzed and compared the lengths of 191 *bla*_OXA-48_-like-harboring plasmids of *K. pneumoniae* and compared the lengths of plasmids harboring different variants of *bla*_OXA-48_-like genes. The lengths of the 191 *bla*_OXA-48_-like-harboring plasmids of *K. pneumoniae* ranged from 5.85 to 337.0 kb, and the 25th percentile, median, and 75th percentile were 6.14 kb, 63.59 kb, and 65.50 kb, respectively (**Figure 1B**). The lengths of 102 plasmids harboring *bla*_OXA-48_ ranged from 46.89 to 323.1 kb (25th percentile, 63.59 kb; 75th percentile, 67.10 kb), with a median size of 63.59 kb (**Figure 1B**). For the 22 plasmids harboring *bla*_OXA-181_, their genome sizes ranged from 5.92 to 293.7 kb. The 25th percentile, median, and 75th percentile were 5.92 kb, 28.47 kb, and 51.48 kb, respectively (**Figure 1B**). For the 59 plasmids harboring *bla*_OXA-232_, their genome sizes ranged from 5.85 to 337 kb, and 42 of these 59 were found to be the small plasmids with a length of 6.14 kb (**Figure 1B**). In addition, the length of all five *bla*_OXA-245_-harboring plasmids was 65.93 kb (**Figure 1B**).

### 3.3 Replicon types of *bla*_OXA-48_-like-harboring plasmids of *K. pneumoniae*

**Figure 2:**
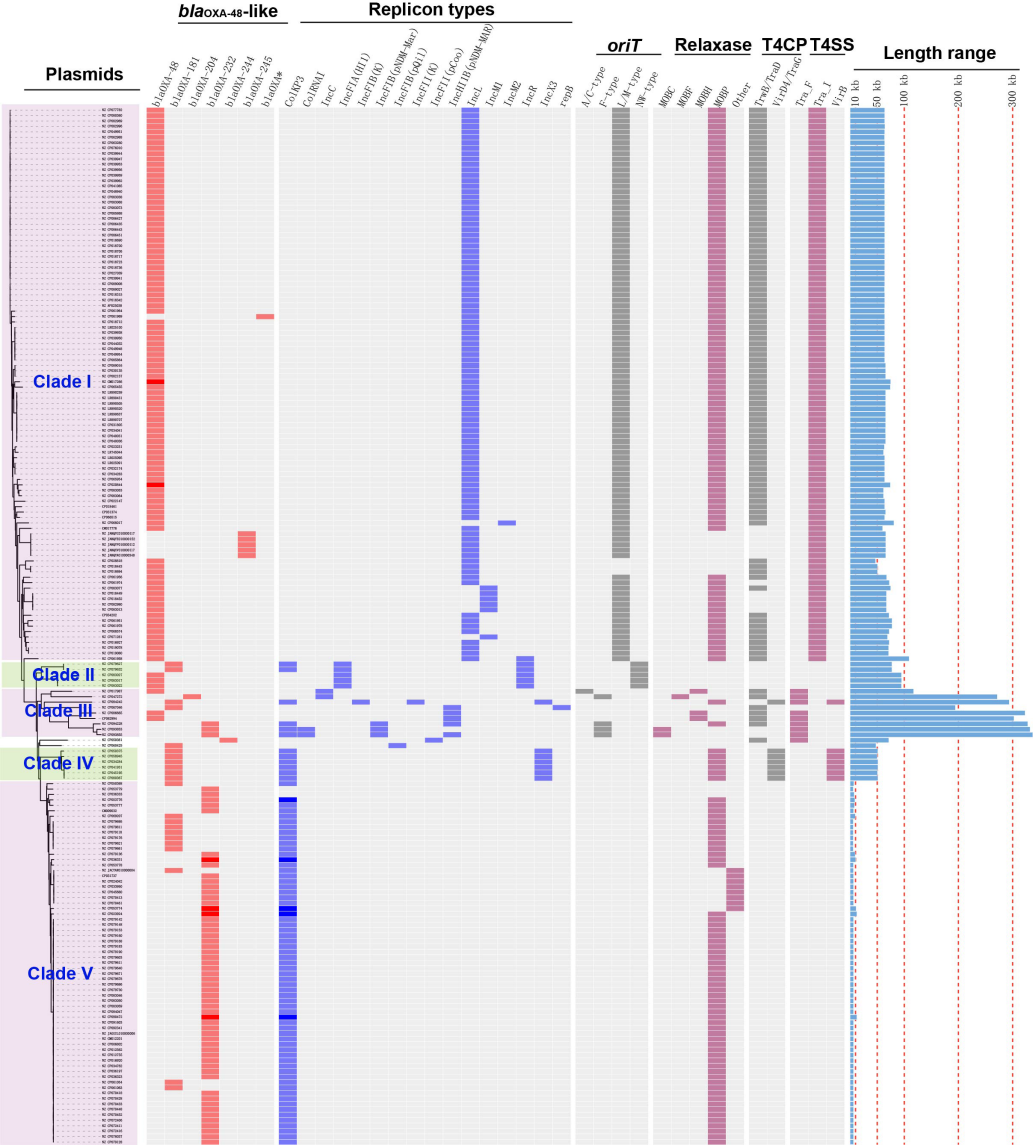
Details of variants of *bla*_OXA-48_-like genes, replicon types, conjugative transfer regions, and length distribution of the 191 *bla*_OXA-48_-like-harboring plasmids of *K. pneumoniae*. The four categories of information present in this figure include the phylogenetic tree, variants of *bla*_OXA-48_-like, replicon types, conjugative transfer regions (*oriT*, relaxase, T4CP, and T4SS), and length distribution of the 191 *bla*_OXA-48_-like-harboring plasmids of *K. pneumoniae*.

Of the 191 *bla*_OXA-48_-like-harboring plasmids, the replicon types of 189 were successfully identified, including 167 single-replicon and 22 multi-replicon plasmids (16 plasmids containing two replicons, four plasmids containing three replicons, and two plasmids containing four replicons) (**Figure 2**).

Among the 167 single-replicon plasmids harboring *bla*_OXA-48_-like in *K. pneumoniae*, plasmids with IncL replicon were the most common, with a total of 94 plasmids (**Figure 2**). In addition, 60 single-replicon plasmids with ColKP3 replicon were selected from the 167 single-replicon *bla*_OXA-48_-like-positive plasmids, which were the second most prevalent single-replicon plasmids harboring *bla*_OXA-48_-like genes in *K. pneumoniae* (**Figure 2**).

In summary, 77 of the 191 *bla*_OXA-48_-like-harboring plasmids of *K. pneumoniae* were found to carry the ColKP3 replicon, accounting for 40.31% of all *bla*_OXA-48_-like-harboring plasmids of *K. pneumoniae* in this study (**Figure 2**). Furthermore, 102 plasmids harboring *bla*_OXA-48_-like genes were classified as IncL/M-type plasmids, with replicon types including IncL, IncM1, or IncM2 (**Figure 2**).

### 3.4 Genetic diversity of the *bla*_OXA-48_-like-harboring plasmids in *K. pneumoniae*

We constructed a phylogenetic cladogram of the 191 *bla*_OXA-48_-like-harboring plasmids to obtain a comprehensive overview of *bla*_OXA-48_-like-harboring plasmid genes in *K. pneumoniae* (**Figure 2**). Based on the phylogenetic cladogram combined with the information on plasmid types, conjugative transfer regions, and genome sizes of the *bla*_OXA-48_-like-harboring plasmids, most of the 191 *bla*_OXA-48_-like-harboring plasmids were clustered into five main clades (clades I–V), representing five *bla*_OXA-48_-like-harboring plasmid patterns in *K. pneumoniae*.

#### 3.4.1 Clade I: IncL/M-type plasmids harboring *bla*_OXA-48_ and *bla*_OXA-245_ in *K. pneumoniae*

**Figure 3:**
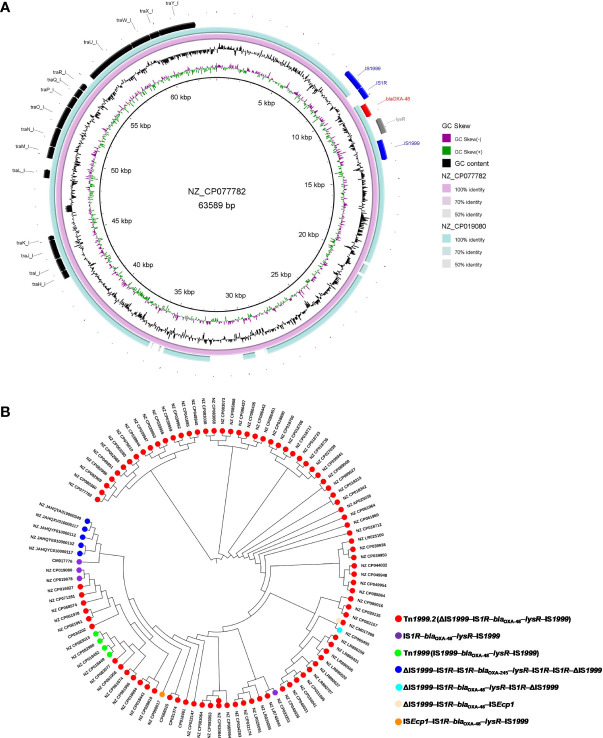
An overview of the conjugative transfer regions and genetic environment surrounding *bla*_OXA-48_ or *bla*_OXA-245_ carried by the 102 IncL/M-type plasmids classified into the clade I cluster. **(A)** Details of the Tra_I-like T4SS gene clusters, transposon Tn*1999.2*, and Tn*1999* in the representative plasmids. **(B)** Distribution of different genetic contexts surrounding *bla*_OXA-48_-like genes in the 102 IncL/M-type plasmids.

**Figure 4:**
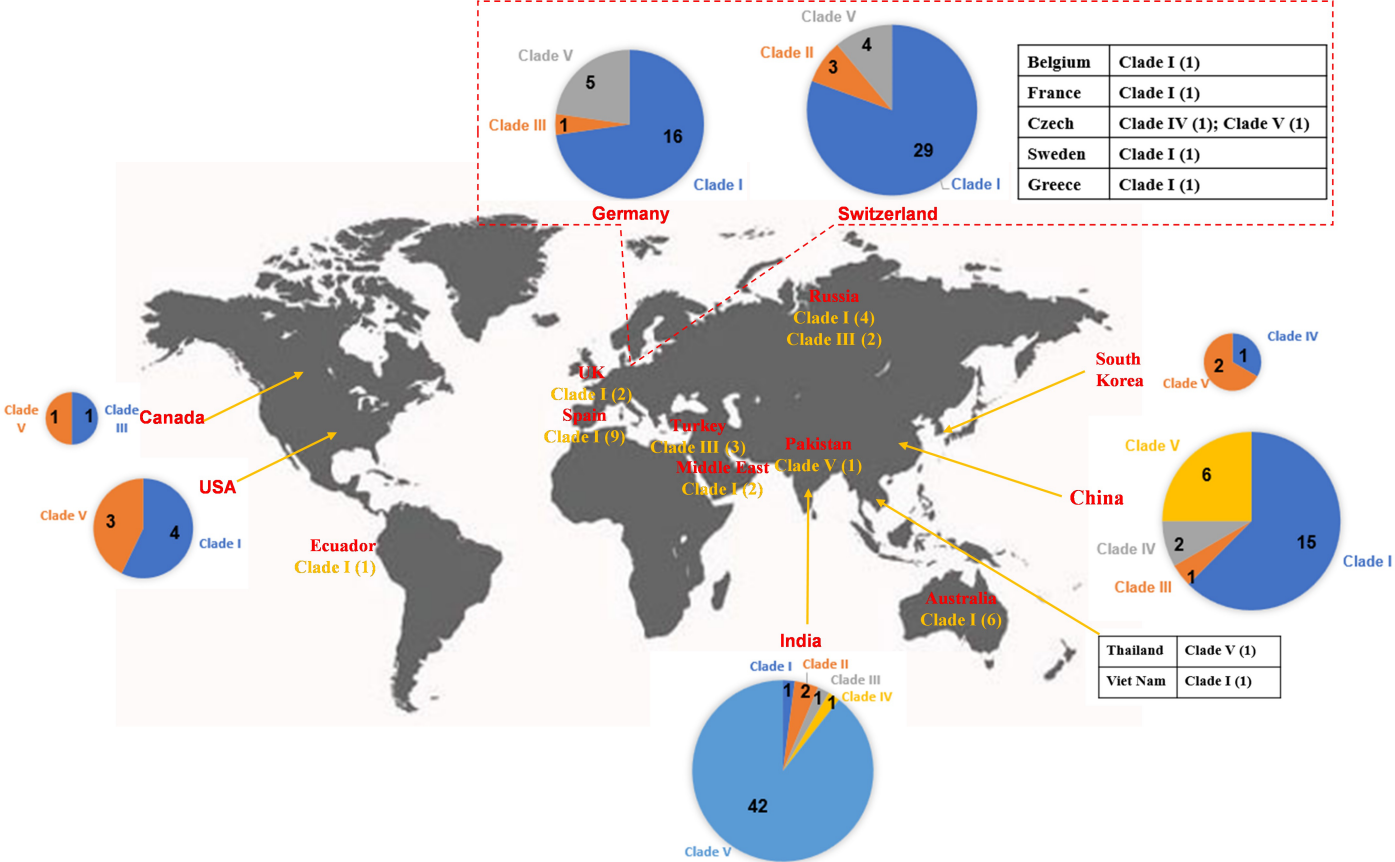
Worldwide distribution of *bla*_OXA-48_-like-harboring plasmids in *K. pneumoniae*. The geographical distribution of the five clades (Clade I–Clade V) from the *bla*_OXA-48_-like-harboring plasmids of *K. pneumoniae* was calculated and displayed by pie chart, text description, as well as tabular form.

A total of 102 IncL/M-type plasmids were classified into the clade I cluster, mainly *bla*_OXA-48_-carrying plasmids (**Figure 2**), accounting for 53.4% of all *bla*_OXA-48_-like-harboring plasmids of *K. pneumoniae*. Most of the 102 IncL/M-type plasmids were single ARG-harboring plasmids (**Figure S1**). Notably, the five bla_OXA-245_-harboring plasmids were also classified into the clade I cluster. The most frequent replicon type of *bla*_OXA-48_-like-harboring IncL/M-type plasmids was the IncL replicon, followed by IncM1 and IncM2. For the 102 IncL/M-type plasmids harboring *bla*_OXA-48_-like genes, their genome sizes ranged from 46.89 to 109.1 kb (25th percentile, 63.59 kb; 75th percentile, 65.68 kb), with a median size of 63.59 kb (**Figure S2**). For the conjugative transfer regions, most of the plasmids of clade I were found to carry L/M-type *oriT*s, genes encoding relaxases of the MOB_P_ family characterized by the domain “Relaxase (Pfam: PF03432),” genes encoding T4CPs of TrwB/TraD subfamily characterized by the domain “TrwB_AAD_bind (PF10412),” and Tra_I-like T4SS gene clusters (**Figures 2**, **3A**), inferred as conjugative plasmids. Members of clade I were widely geographically distributed all over the world, mainly in Switzerland (29 plasmids), Germany (16 plasmids), and China (15 plasmids) (**Figure 4**). Most of the IncL/M-type plasmids were human origins, some were animal origins (collected in Switzerland) and environment origins (also collected in Switzerland) (**Figure S1** and **Table S3**).

We explored the genetic environment surrounding *bla*_OXA-48_-like genes carried by the 102 IncL/M-type plasmids in *K. pneumoniae*. For *bla*_OXA-48_-carrying IncL/M-type plasmids in *K. pneumoniae*, the *bla*_OXA-48_ was mainly located on the composite transposon Tn*1999.2* (ΔIS*1999*–IS*1R*–*bla*_OXA-48_–*lysR*–IS*1999*), with 87 plasmids carrying intact Tn*1999.2* and four plasmids harboring the truncated Tn*1999.2* (IS*1R*–*bla*_OXA-48_–lysR–IS*1999*) (**Figure 3A**). We also found four *bla*_OXA-48_-harboring plasmids of clade I carrying classical Tn*1999* (IS*1999*–*bla*_OXA-48_–lysR–IS*1999*). Notably, several *bla*_OXA-48_-harboring plasmids were found to carry specific genetic contexts of *bla*_OXA-48_, including *K. pneumoniae* strain KPN1482 plasmid pKPN1482-3 (NZ_CP020844) with two copies of intact Tn*1999.2* (ΔIS*1999*–IS*1R*–*bla*_OXA-48_–*lysR*–IS*1999*), *K. pneumoniae* strain F64 plasmid pRYC-OXA48 (NZ_CM017266) carrying the intact Tn*1999.2* (ΔIS*1999*–IS*1R*–*bla*_OXA-48_–*lysR*–IS*1999*) and the structure “ΔIS*1999*–IS*1R*–*bla*_OXA-48_–IS*Ecp1*”, *K. pneumoniae* strain Beach Ranger plasmid pBR_02 (NZ_CP065455) carrying the “ΔIS*1999*–IS*1R*–*bla*_OXA-48_–*lysR*–IS*1R*–ΔIS*1999*”, as well as the *K. pneumoniae* strain 2016_49 plasmid pMS3802OXARMA (NZ_CP068017) carrying the structure “IS*Ecp1*–IS*1R*–*bla*_OXA-48_–*lysR*–IS*1999*” (**Figure S2**). In addition, we found that the five *bla*_OXA-245_-harboring IncL plasmids, which were all isolated in Spain, carried the structure “ΔIS*1999*–IS*1R*–IS*1R*–*bla*_OXA-245_–*lysR*–IS*1R*–IS*1R*–ΔIS*1999*” (**Figure 3B**).

#### 3.4.2 Clade II: Mobilizable *bla*_OXA-48_-like-harboring plasmids in *K. pneumoniae*

Five *bla*_OXA-48_-like-harboring multidrug-resistant (MDR) plasmids with IncFIA(HI1) and IncR replicons were classified into a small cluster, clade II of the phylogenetic cladogram constructed with the 191 *bla*_OXA-48_-like-harboring plasmids, including three 94.9-kb plasmids [IncFIA(HI1):IncR] harboring *bla*_OXA-48_ and two 77.1-kb plasmids [ColKP3:IncFIA(HI1):IncR] harboring *bla*_OXA-181_ (**Figure 2**). These five plasmids all carried NW-type *oriT*s, but not genes encoding relaxase, T4CP, or T4SS in their genomes, indicating that they are mobilizable plasmids (**Figure 2**). The five plasmids belonging to clade II were found to be distributed in Switzerland and India (**Figure 4**), which were all human origins (**Figure S1** and **Table S3**).

For the three *bla*_OXA-48_-harboring plasmids isolated in Switzerland, *bla*_OXA-48_ was situated in the truncated Tn*1999.2* (IS*1R*–*bla*_OXA-48_–*lysR*–IS*1999*) (**Figure S3A**). For the two *bla*_OXA-181_-harboring plasmids isolated in India, intact IS*3000* was located upstream of *bla*_OXA-181_ (**Figure S3B**).

#### 3.4.3 Clade III: Mega *bla*_OXA-48_-like-harboring plasmids in *K. pneumoniae*

One cluster of the phylogenetic cladogram (clade III) composed of nine mega MDR plasmids with lengths ranging from 117.2 to 337.0 kb (25th percentile, 233.1 kb, median, 302.4 kb, and 75th percentile, 330.1 kb) was identified, including *bla*_OXA-48_-harboring, *bla*_OXA-181_-harboring, *bla*_OXA-204_-harboring, and *bla*_OXA-232_-harboring plasmids (**Figures 2**, **S2**). The plasmids in clade III contained four single-replicon plasmids, one plasmid with two different replicons, two plasmids with three different replicons, and two plasmids with four different replicons (**Figure 2**). Among the nine mega plasmids, the IncHI1B(pNDM-MAR) and ColKP3 replicons were relatively common, with the IncHI1B(pNDM-MAR) replicon found in five plasmids and the ColKP3 replicon found in four plasmids (**Figure 2**). Most of the plasmids belonging to clade III had the genes encoding for T4CPs of TrwB/TraD subfamily characterized by the domain “TrwB_AAD_bind (PF10412)” and Tra_F-like T4SS gene clusters (**Figure 2**). According to the identified conjugative transfer regions, these mega plasmids were inferred as conjugative plasmids. However, they were heterogeneous in the type of relaxase genes, and only five plasmids had their *oriT*s successfully identified using oriTfinder. The plasmids of clade III were sporadically discovered in Turkey, Russia, China, India, Germany, and Canada (**Figure 4**), originated from samples of human (**Figure S1** and **Table S3**).

For the three *bla*_OXA-48_-harboring plasmids, including one 117.2-kb IncC plasmid and two IncHI1B(pNDM-MAR) plasmids with lengths >300 kb, *bla*_OXA-48_ was found to be situated in the truncated Tn*1999.2* (IS*1R*–*bla*_OXA-48_–*lysR*–IS*1999*). For the two *bla*_OXA-181_-harboring plasmids, one 293-kb plasmid carried the genetic environment of *bla*_OXA-181_ (IS*26*–ΔIS*3000*–ΔIS*Ecp1*–*bla*_OXA-181_–Δ*lysR*–Δ*ere*–Δ*repA*–IS*Kpn19*), and another 194-kb plasmid carried the “IS*Ecp1*–*bla*_OXA-181_”. For the *bla*_OXA-204_-harboring plasmid and the three *bla*_OXA-232_-harboring plasmids, IS*Ecp1* was inserted upstream of *bla*_OXA-204_ or *bla*_OXA-232_.

#### 3.4.4 Clade IV: *bla*_OXA-181_-harboring plasmids with multi-replicon ColKP3:IncX3 in *K. pneumoniae*

**Figure 5:**
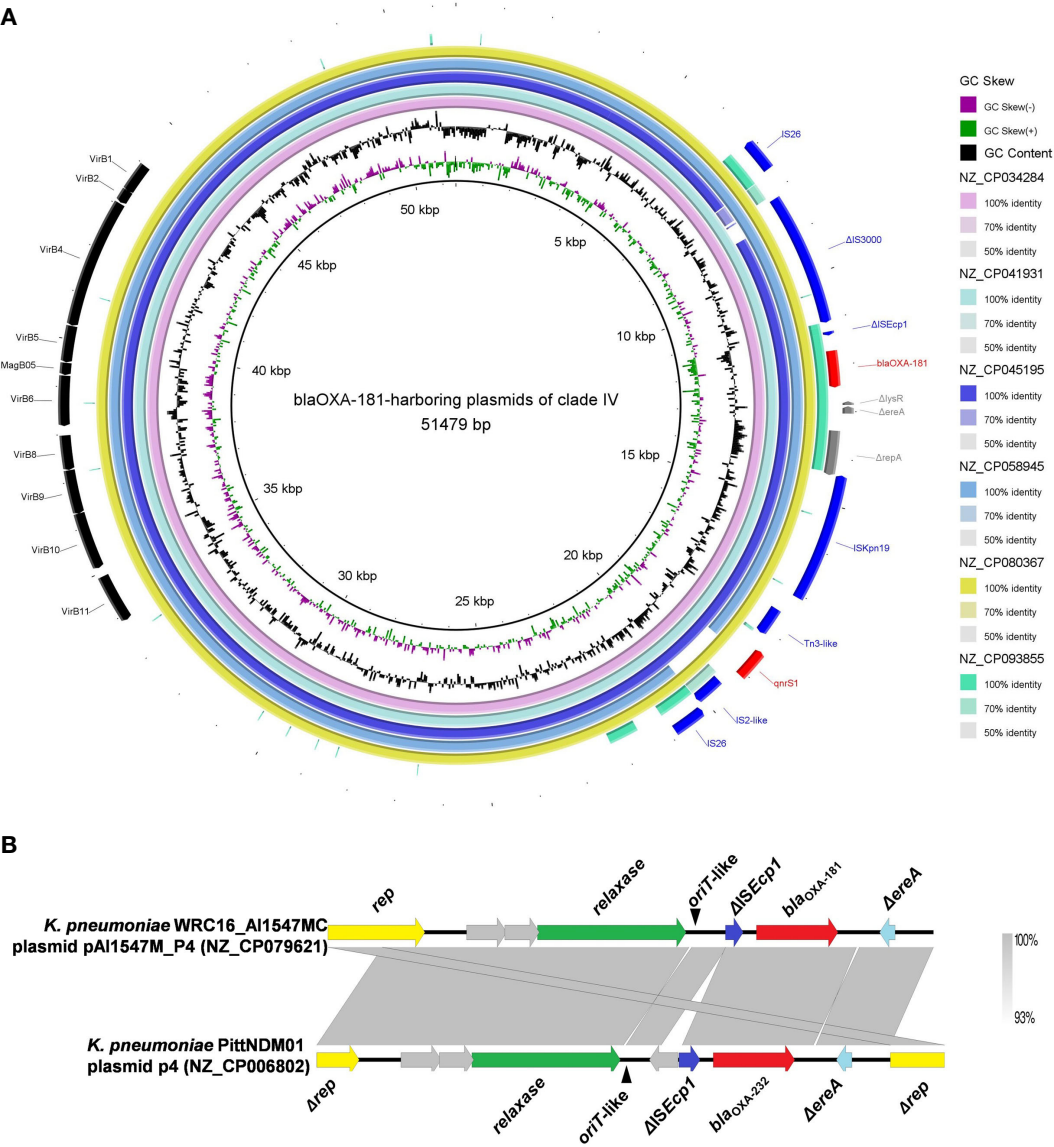
Characteristics of the conjugative transfer regions and genetic environment surrounding *bla*_OXA-48-like_ carried by the plasmids belonging to clade IV and clade V. **(A)** Details of the VirB-like T4SS gene clusters and *bla*_OXA-181_-associated genetic structures identified among the six *bla*_OXA-181_-harboring plasmids clustered into clade IV. **(B)** The *oriT*-like regions and the genetic context surrounding the *bla*_OXA-181_ or *bla*_OXA-232_ genes of the small plasmids belonging to clade V.

Six *bla*_OXA-181_-harboring plasmids with multi-replicon ColKP3:IncX3 were clustered into clade IV, and their genome sizes ranged from 50.13 to 51.48 kb (**Figure 2**). Almost all the plasmids of clade IV harbored two ARGs: *bla*_OXA-181_ and *qnrS1* (**Figure S1**). They all carried genes encoding relaxases of the MOB_P_ family characterized by the domain “Relaxase (PF03432),” genes encoding T4CPs of the VirD4/TraG subfamily characterized by the domain “T4SS-DNA_transf (PF02534),” and mostly VirB-like T4SS gene clusters (**Figures 2**, **5A**). Although we could not determine the definite *oriT* sites of the clade IV plasmids, the *oriT*-like region flanking the relaxase genes was found in all six *bla*_OXA-181_-harboring plasmids, characterized by the inverted repeat (IR) sequence (TAACTA.TAGTTA). According to the identified conjugative transfer regions, the plasmids of clade IV should be conjugative plasmids. The plasmids of clade IV (human origins) were sporadically discovered in China, South Korea, India, and Czech Republic (**Figures 4**, **S1**; **Table S3**).

For the six ColKP3-IncX3 hybrid plasmids harboring both *bla*_OXA-181_ and *qnrS1* in their genomes, *bla*_OXA-181_ and *qnrS1* were found to be located in a composite transposon, which was bracketed by two copies of the insertion sequence IS*26* in the same orientation (IS*26*–ΔIS*3000*–ΔIS*Ecp1*–*bla*_OXA-181_–Δ*lysR*–Δ*ere*–Δ*repA*–IS*Kpn19*–Tn*3*-like–*qnrS1*–IS*2*-like–IS*26*) (**Figure 5A**).

#### 3.4.5 Clade V: Small mobilizable *bla*_OXA-48_-like-harboring plasmids in *K. pneumoniae*

A total of 67 small plasmids with lengths ranging from 5.85 to 12.27 kb (mostly 6.14 kb), including 11 *bla*_OXA-181_-harboring plasmids and 56 *bla*_OXA-232_-harboring plasmids, were grouped into a large cluster named clade V of the phylogenetic cladogram (**Figure 2**). Almost all the plasmids classified into clade V were single ARG-harboring plasmids (**Figure S1**). Most of the 67 small plasmids belonging to clade V were identified as single-replicon plasmids with a ColKP3 replicon. Moreover, most of the plasmids belonging to clade V carried genes encoding relaxases of the MOB_P_ family characterized by the domain “Relaxase (Pfam: PF03432),” but no gene encoding T4CP or T4SS (**Figures 2**, **5B**). Although no *oriT* was identified in the plasmids of clade V, the *oriT*-like regions adjacent to relaxase genes were identified with an IR sequence (AAAAGGAAAGTG.CACTTTCCTTTT) (**Figure 5B**). According to the identified conjugative transfer regions, the plasmids of clade V should be mobilizable plasmids. Among the 67 plasmids belonging to clade V, 42 (62.69%) were geographically found in India (**Figure 4**). These ColKP3-type mobilizable plasmids were also found in China, Germany, Switzerland, the USA*, etc* (**Figure 4**). Notably, the ColKP3-type mobilizable plasmids were found to be human origins (**Figure S1** and **Table S3**).

We explored the genetic context surrounding the *bla*_OXA-181_ or *bla*_OXA-232_ genes of the 67 small plasmids belonging to clade V. For the 67 small ColKP3-type plasmids belonging to clade V, *bla*_OXA-181_ or *bla*_OXA-232_ were located downstream of the ΔIS*Ecp1* harbored by the ColKP3-type plasmid (**Figure 5B**).

## 4 Discussion

OXA-48-like carbapenemases are important causes of non-susceptibility to carbapenems in *Enterobacterales* (Pitout et al., 2019). *bla*_OXA-48_-like genes are always plasmid-borne, and plasmids make considerable contributions to disseminating *bla*_OXA-48_-like genes (de Jonge et al., 2016). To characterize plasmids harboring *bla*_OXA-48_-like genes in *K. pneumoniae*, we systematically analyzed the variants of *bla*_OXA-48_-like, replicon types, conjugative transfer regions, and genetic contexts of *bla*_OXA-48_-like plasmids among 191 *bla*_OXA-48_-like-harboring plasmids, which were selected from 4451 plasmids belonging to *K. pneumoniae* from the NCBI GenBank database. In our study, seven different variants of *bla*_OXA-48_-like genes were identified from 191 *bla*_OXA-48_-like-harboring plasmids in *K. pneumoniae*, with *bla*_OXA-48_, *bla*_OXA-232_, and *bla*_OXA-181_ being highly prevalent.

The *bla*_OXA-48_-carrying plasmids were the most prevalent, accounting for 53.40% of the 191 *bla*_OXA-48_-like-harboring plasmids in *K. pneumoniae*. Currently, OXA-48 is the most common OXA-48-like carbapenemase worldwide; it was first reported in 2004 on a 70-kb plasmid of *K. pneumoniae* isolated in Turkey (Poirel et al., 2004). After the first report, the presence of OXA-48 was reported in many members of *Enterobacterales* (Pitout et al., 2019). Our results showed that IncL/M-type conjugative plasmids were important carriers of *bla*_OXA-48_ in *K. pneumoniae*, mainly IncL plasmids, followed by IncM1 and IncM2 plasmids. The broad-host-range IncL/M-type plasmids are now frequently found in environmental and clinical strains (Potron et al., 2013; Woerther et al., 2018), which have been demonstrated as contributors to the dissemination of genes encoding broad-spectrum β-lactam resistance, including *bla*_OXA-48_ (Carrër et al., 2008) *bla*_NDM-1_ (Aubert et al., 2003), and *bla*_CTX-M-3_ (Oteo et al., 2015). The spread of *bla*_OXA-48_ is largely driven by Tn*1999* and its variants, which are situated on pOXA-48a-like IncL/M-type conjugative plasmids (Potron et al., 2013). In our study of *bla*_OXA-48_-carrying IncL/M-type plasmids in *K. pneumoniae*, the *bla*_OXA-48_ was mainly located on the composite transposon Tn*1999.2*, a variant of Tn*1999*. Tn*1999* contains two copies of IS1999; one copy is located upstream of *bla*_OXA-48_, and another is situated downstream of *bla*_OXA-48_–*lysR*. IS*1999* was first reported in *Pseudomonas aeruginosa* isolates from Thailand and was inserted into the integron-specific recombination site *attI1* upstream of *bla*_VEB-1_ (Aubert et al., 2003). Tn*1999.2*, first described in Turkey from 2006 to 2007, is a Tn*1999* variant with an IS*1R* inserted into IS*1999* upstream of *bla*_OXA-48_, generating a strong hybrid promoter, resulting in higher enzymatic activity than that of Tn*1999* (Carrër et al., 2008).

In our study, *bla*_OXA-232_-harboring plasmids were the second most common plasmids carrying *bla*_OXA-48_-like genes in *K. pneumoniae*, accounting for 30.89% of the 191 *bla*_OXA-48_-like-harboring plasmids. The variant *bla*_OXA-232_ was first found in 2012 in *K. pneumoniae* and *E. coli* isolates obtained from French patients who had traveled to India (Potron et al., 2013). In China, the *bla*_OXA-232_ was first reported in 2017 in *K. pneumoniae* (Yin et al., 2017). In *K. pneumoniae*, the *bla*_OXA-232_ gene was mainly carried by 6.1-kb ColKP3-type mobilizable plasmids. These small ColKP3-type mobilizable plasmids harboring *bla*_OXA-232_ carried *oriT*-like regions characterized by the IR sequence (AAAAGGAAAGTG.CACTTTCCTTTT) and relaxases of the MOB_P_ family characterized by the domain “Relaxase (Pfam: PF03432),” with TraI protein encoded by the IncPα plasmid RP4 (Pansegrau et al., 1993) as a representative.

In this study, *bla*_OXA-181_ was another common variant in *K. pneumoniae*, and plasmids harboring *bla*_OXA-181_ accounted for 11.52% of the 191 *bla*_OXA-48_-like-harboring plasmids. OXA-181 was first identified in CRKP and *Enterobacter cloacae* strains isolated from Indian hospitals in 2007 (Castanheira et al., 2011). Since then, OXA-181-producing *Enterobacterales*, mainly *K. pneumoniae* and *Escherichia coli*, have been reported in several countries worldwide (Balm et al., 2013; Liu et al., 2015; Rojas et al., 2017; Piazza et al., 2018; Mouftah et al., 2019; Liu et al., 2020). Four plasmid types belonging to ColKP3, IncX3, IncT, and IncN1 replicons have been reported to harbor the OXA-181 gene (*bla*_OXA-181_) (Pitout et al., 2019). Our study showed that the *bla*_OXA-181_-harboring plasmids mainly included two categories in *K. pneumoniae*: one was a group of 50-kb ColKP3-IncX3 hybrid conjugative plasmids, and the other was a group of small ColKP3-type mobilizable plasmids with lengths of 5.9–9.3 kb.

Notably, five *bla*_OXA-245_-harboring IncL plasmids with a length of 65.9 kb in *K. pneumoniae* were found in our analysis, which were all isolated from Spain. OXA-245, differing from OXA-48 in a single amino acid substitution (Glu125Tyr), was first identified in 2011 in a *K. pneumoniae* isolate collected in Spain (Pérez-Vázquez et al., 2016). In Spain, OXA-245 has been closely related to *K. pneumoniae* ST11 (Oteo et al., 2015) and also exhibited co-production of CTX-M-15 (Pérez-Vázquez et al., 2016). Similar to *bla*_OXA-48_, *bla*_OXA-245_ was located in the variant Tn*1999* and carried by a 60-kb IncL/M-type plasmid. However, the genetic context of *bla*_OXA-245_ carried by the five 65.9-kb IncL plasmids was more complicated than that of *bla*_OXA-48_-harboring IncL/M-type plasmids, with two copies of IS*1R* inserted upstream and downstream of *bla*_OXA-245_–*lysR*.

## 5 Conclusion

In this study, we analyzed the variants of *bla*_OXA-48_-like, replicon types, phylogenetic patterns, geographic distribution, conjugative transfer regions, and the genetic environments surrounding *bla*_OXA-48_-like of 191 *bla*_OXA-48_-like-harboring plasmids, which were identified from 4451 plasmids of *K. pneumoniae* downloaded from GenBank. Seven variants of *bla*_OXA-48_-like were found among the 191 *bla*_OXA-48_-like-harboring plasmids, with *bla*_OXA-48_, *bla*_OXA-232_, and *bla*_OXA-181_ as the most dominant. The *bla*_OXA-48_ was mainly harbored by the composite transposon Tn*1999.2* located on IncL/M-type conjugative plasmids, which were mainly geographically distributed in Switzerland, Germany, and China. The *bl*a_OXA-232_ was mainly carried by 6.1-kb ColKP3-type mobilizable plasmids, which were mainly geographically distributed in India. The *bla*_OXA-181_ was mainly carried by a group of 50-kb ColKP3-IncX3 hybrid conjugative plasmids (sporadically discovered in China, South Korea, India, and Czech Republic) and a group of small ColKP3-type mobilizable plasmids with lengths of 5.9–9.3 kb (mainly isolated in India). In addition, five *bla*_OXA-245_-harboring 65.9-kb IncL plasmids in *K. pneumoniae* (isolated in Spain) were found to have the genetic context of *bla*_OXA-245_ more complicated than that of *bla*_OXA-48_-harboring IncL/M-type plasmids. This study provides important insights into the phylogeny and evolution of *bla*_OXA-48_-like-harboring plasmids in *K. pneumoniae* and further addresses their role in the acquisition and spread of resistance genes.

## Data availability statement

The datasets presented in this study can be found in online repositories. The names of the repository/repositories and accession number(s) can be found in the article/**Supplementary Material**.

## Author contributions

XL and WL conceived and designed the project. WL, HG and XL analysed all the data and wrote the manuscript. YG, XYa, RL, and SL performed data acquisition. CS, WD, and SC provided the technical assistance. PX and WH provided some suggestions for manuscript writing. XL, XYi and JS reviewed and edited the manuscript. All authors read and approved the final manuscript. All authors contributed to the article and approved the submitted version.
