# Supplementary material for: Comparative genomic analysis of plasmids harboring bla OXA-48-like genes in Klebsiella pneumoniae
Source: Front Cell Infect Microbiol. 2022 Dec 20;12:1082813. doi: 10.3389/fcimb.2022.1082813 (PMC9807924; doi:10.3389/fcimb.2022.1082813)
Supplement: Supplementary file 1 [file DataSheet_1.docx]

**Comparative Genomic Analysis of Plasmids Harboring *bla*_OXA-48_-like Genes in *Klebsiella pneumoniae***

**Table legends：**

**Table S1.** Detailed information of 4451 plasmids of *K. pneumoniae* downloaded from the GenBank database.

**Table S2.** Details of acquired antibiotic resistance genes for the *bla*_OXA-48_-like-harboring plasmids of *K. pneumoniae* using software ResFinder.

**Table S3.** Detailed information about “isolation_source” and “host” of the 191 *bla*_OXA-48_-like-harboring plasmids in *K. pneumoniae.*

**Figure legends:**

**Figure S1**. Details of ARGs, geographic distribution, and collection date of the 191 *bla*_OXA-48_-like-harboring plasmids of *K. pneumoniae*. All the ARGs carried by the 191 *bla*_OXA-48_-like-harboring plasmids could be classified into ten classes. Information about “isolation_source” and “host” of the 191 *bla*_OXA-48_-like-harboring plasmids of *K. pneumoniae* were also marked in the image.

**Figure S2**. Length distribution of the *bla*_OXA-48_-like-harboring plasmids belonging to clades I–III.

**Figure S3**. Genetic contexts surrounding *bla*_OXA-48_-like genes of the three *bla*_OXA-48_-harboring plasmids isolated in Switzerland (A) and the two *bla*_OXA-181_-harboring plasmids isolated in India (B) belonging to clade II.

Figure S
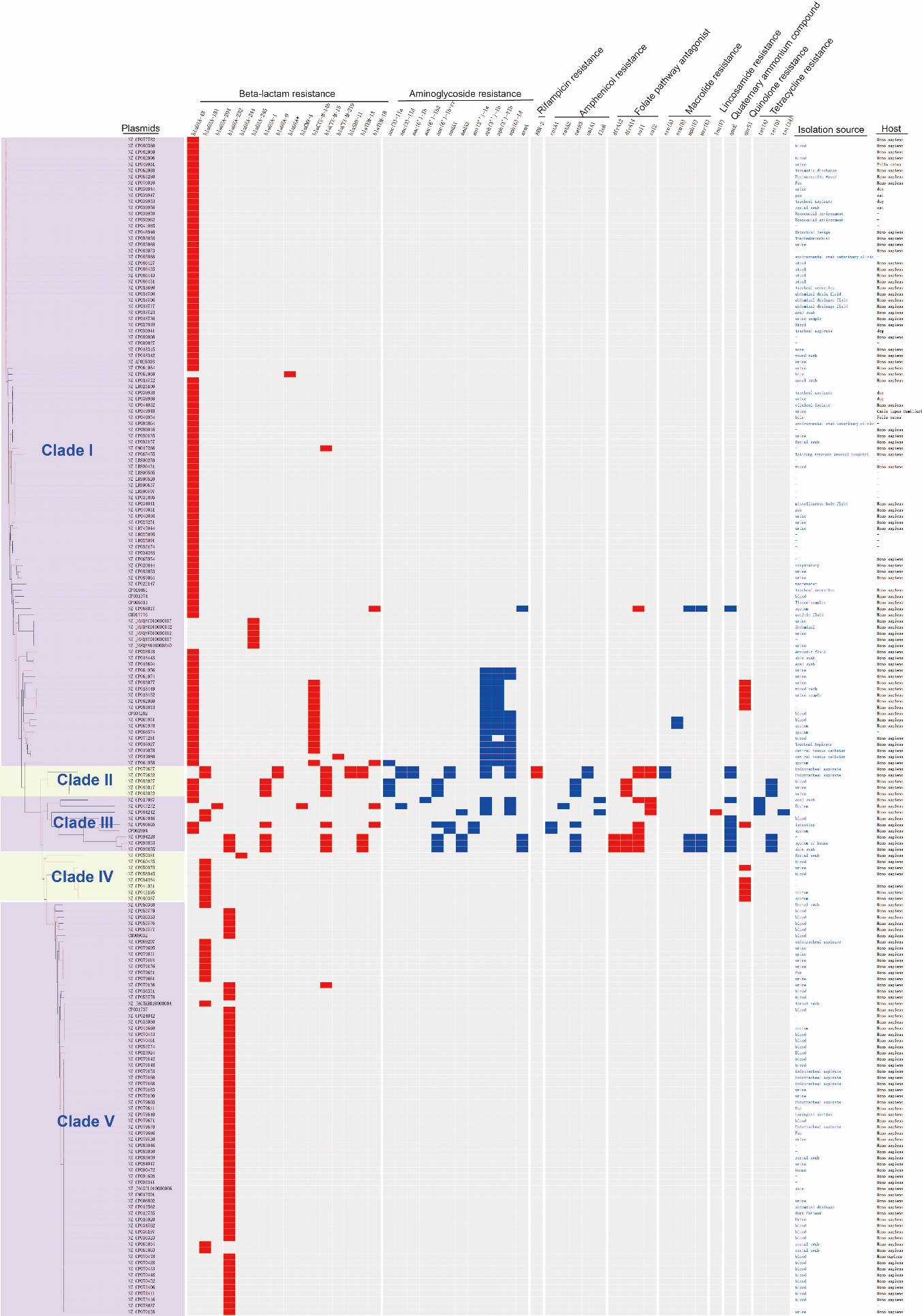
1

Figure S2


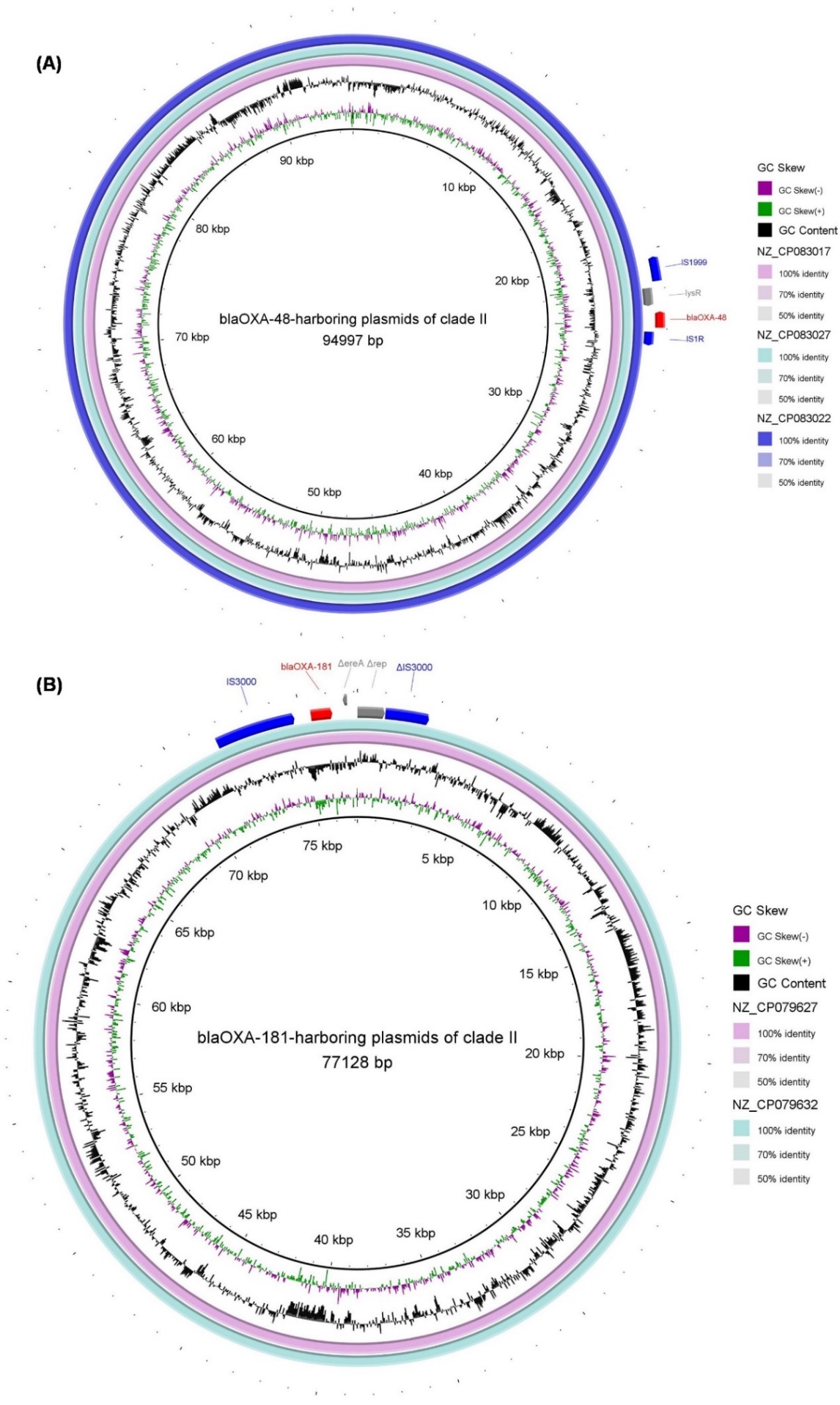


Figure S3
